# Enhanced surveillance for tick-borne rickettsiosis and ehrlichiosis in North Carolina: Protocol and preliminary results

**DOI:** 10.1371/journal.pone.0320361

**Published:** 2025-05-12

**Authors:** Lauryn Ursery, Odai Mansour, Haley Abernathy, Emily Wichmann, Allie Yackley, Alexis Siegler, Dana Giandomenico, Carl Williams, Alexis Barbarin, Michael H. Reiskind, Ross M. Boyce

**Affiliations:** 1 Institute for Global Health and Infectious Diseases, University of North Carolina at Chapel Hill, Chapel Hill, North Carolina, United States of America; 2 School of Medicine, University of North Carolina at Chapel Hill, Chapel Hill, North Carolina, United States of America; 3 Department of Epidemiology, Gillings School of Global Public Health, University of North Carolina at Chapel Hill, Chapel Hill, North Carolina, United States of America; 4 Department of Entomology and Plant Pathology, North Carolina State University, Raleigh, North Carolina, United States of America; 5 College of Arts & Sciences, University of North Carolina at Chapel Hill, Chapel Hill, North Carolina, United States of America; 6 Division of Public Health, North Carolina Department of Health and Human Services, Raleigh, North Carolina, United States of America; 7 Carolina Population Center, University of North Carolina at Chapel Hill, Chapel Hill, North Carolina, United States of America; The University of Toledo College of Medicine: The University of Toledo College of Medicine and Life Sciences, UNITED STATES OF AMERICA

## Abstract

North Carolina (NC) experiences some of the highest incidence rates of spotted fever rickettsiosis (SFR) and ehrlichiosis in the United States (US). Due to the non-specific nature of clinical symptoms, minimal utilization of molecular methods when appropriate, and limitations of sero-diagnostic methods, accurate case identification and subsequent public health reporting is challenging. Herein we detail the protocol and early enrollment results for an enhanced surveillance project aiming to generate more accurate estimates of tick-borne disease incidence in NC. Secondary outcomes of interest include: (i) increasing the obtainment rate of convalescent samples (ii) defining demographic and socioeconomic, behavioral/knowledge, entomologic, and environmental risk factors for disease, and (iii) describing the spectrum and clinical course of disease among cases of SFR and ehrlichiosis up to 90 days after symptom onset. In addition, we will collect remnant serum to establish a biorepository of well characterized samples that we intend to make available to researchers. Of the 150 participants enrolled, highlighted results include 49.5% of participants reported being exposed in their own home compared to 43.2% being exposed due to work or travel showing the importance of tick control and education. We also reported more confirmed cases of SFR and ehrlichiosis (15 and 20 respectively) where the North Carolina State Health Department only reported 14 and 11 confirmed cases in the entire state in 2022. Findings from the project will be reported in subsequent publications.

## Introduction

Vector-borne diseases (VBD) are an emerging public health problem in the United States (US) [1]. While outbreaks of VBDs such as malaria frequently attract attention [2], the vast majority - approximately 75% - of VBDs reported in the US are tick-borne diseases (TBDs) such as Lyme disease (LD), spotted fever rickettsiosis (SFR), anaplasmosis, babesiosis, and ehrlichiosis. For example, in the most recent year of complete reporting there were nearly 35,000 cases of LD - a number that underestimates the true number of infections [3,4]. Although less frequently reported, the incidence of clinical diseases due to infection with *Ehrlichia* species and *Rickettsia parkeri*, which are transmitted by the lone star (*Amblyomma americanum*) and Gulf Coast (*Amblyomma maculatum*) ticks, respectively, are also increasing, particularly in the southeastern US [5,6]. For example, the annual number of ehrlichiosis cases reported to the Centers for Disease Control and Prevention (CDC) has increased more than ten-fold since 2000 [7].

Despite the increasing rates and evolving geographic risk, an accurate estimate of TBD epidemiology is greatly constrained by limits of serological testing, which despite PCR being a useful tool for acute ehrlichiosis, serologic methods remain the primary means of diagnosis for many of these diseases [8]. While indirect immunofluorescence assays (IFA) are used for TBD, they rely on the detection of antibodies, which may not be present early in the course of illness when most patients seek care. Therefore, guidelines recommend that providers treat suspected patients empirically, but a second, convalescent sample must be drawn 2–10 weeks later to confirm the diagnosis. Yet very few patients complete both acute and convalescent testing, resulting in the vast majority of cases being classified for surveillance purposes at lower levels of certainty (i.e., suspect). In fact, less than 3% of SFR cases reported to the CDC are classified as confirmed [3,9]. Additionally, with studies conducted in endemic areas showing seroprevalence rates upwards of 10–20% in the general population [10], it is difficult to distinguish incident cases from background seroprevalence when only a single serologic result is available.

These issues limit our knowledge of the risk factors associated with many TBDs, specifically regarding exposure to ticks given (i) the low rate of obtaining convalescent samples and (ii) amount of time between exposure and diagnostic confirmation. Even as the seasonality and geographic distribution of ticks is evolving [6], the reasons for increasing number of documented tick-human encounters are not fully elucidated. Existing TBD surveillance systems provide limited information on when and where people are being exposed to ticks [11]. Even when information is available, it is often aggregated, county-level data, which may miss important, but small-scale variations in the ecology of ticks and reservoirs[12].

Therefore, the overarching goal of this enhanced surveillance project supported by North Carolina Department of Health and Human Services, and CDC, was to generate more accurate epidemiological estimates of TBD incidence in NC, while also advancing our knowledge of potential demographic, behavioral, and environmental risk factors associated with infection. To achieve this objective, we implemented an active approach to surveillance comprised of three elements: (i) dedicated follow-up of potential cases to improve completion rates of diagnostic testing algorithms, (ii) utilization of more detailed case report forms provided to potential cases as online surveys, and (iii) home visits to assess environmental and entomological risk within the peri-domestic space. Separate from the surveillance study supported by CDC, the project framework also allowed for the collection of remnant samples (i.e., leftover blood and serum) from clinical laboratories towards the establishment of a biorepository for future research. Herein, we describe the enhanced surveillance protocol and present preliminary findings regarding enrollment and data collection.

## Methods

### Overview

**Fig 1:**
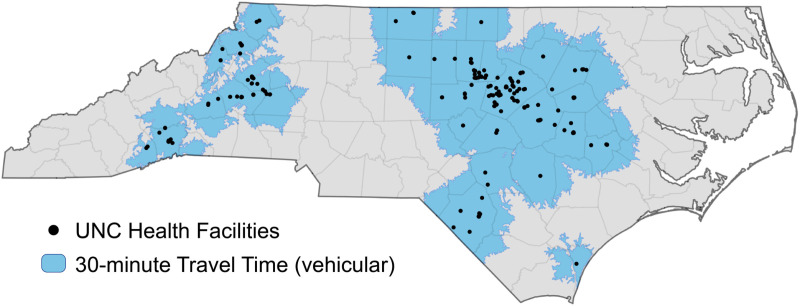
Map of UNC Health System Across North Carolina. [14].

Adult residents of NC who presented to any healthcare facility within the University of North Carolina (UNC) Health system with acute symptoms and had diagnostic testing for SFR and/or ehrlichiosis ordered by the medical provider were eligible to participate. UNC Health (Fig 1) is the largest academic health system in NC. With 14 hospitals and over 500 clinics located across the state, UNC Health reports approximately 3.5 million clinical visits each year [13]. Screening and sample collection began August 23^rd^ 2021, and is still ongoing. Enrollment began on June 26^th^ 2022 and is still ongoing.

**Fig 2:**
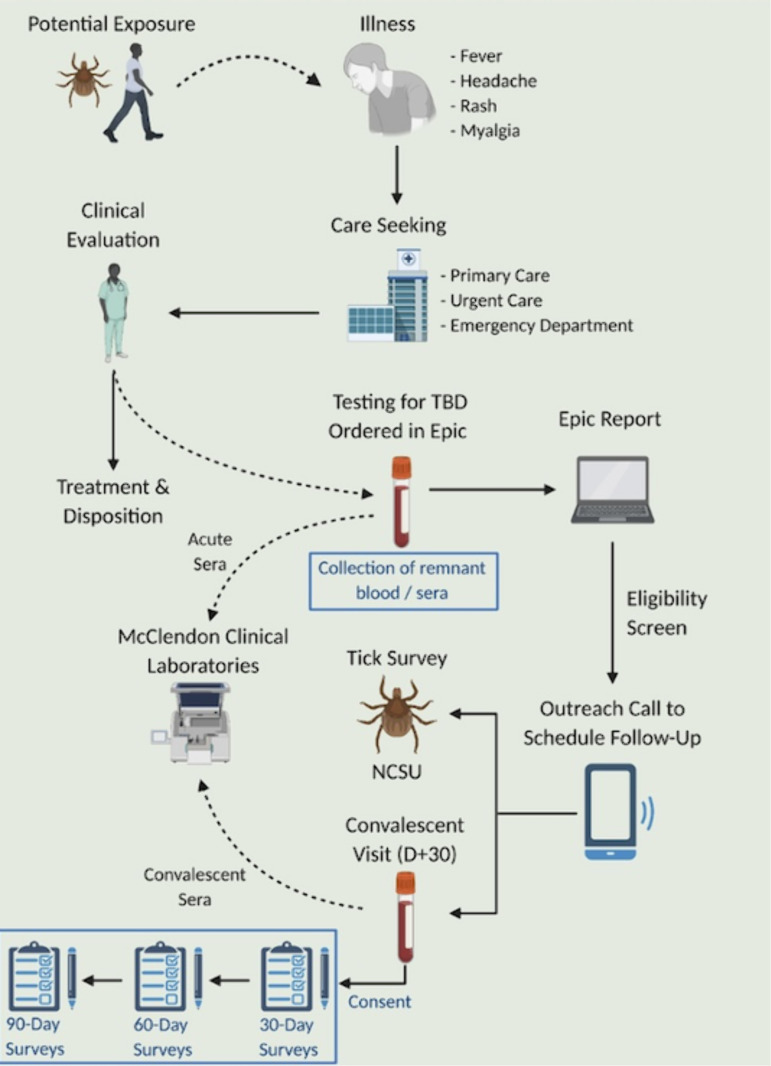
Study Schema. “Reprinted from BioRender under a CC BY license, with permission from BioRender original copyright 2025” [15].

Because all serological samples are sent to McLendon Clinical Laboratories for testing, remnant clinical samples, including those who are not eligible or who did not participate in the study, are able to be collected and stored for future research after routine testing is complete. If available, whole blood samples are collected. The study schema is summarized in Fig 2 while schedules of activities of participants are summarized in Table 1. Further details of each step in the protocol are provided below.

**Table 1:** Summary of Enhanced surveillance activities.

	ENCOUNTER						
**Acute Visit**	**Screening & Enrollment**	**Baseline Survey**	**Convalescent** **Visit**	**Ecological Assessment**	**Recovery Eval 1**	**Recovery Eval 2**
**Timeframe**	**Day 0**	**D + 7**	**D + 10**	**D + 30**	**D + 14**	**D + 60**	**D + 90**
**Encounter Type**							
Routine Care	X						
Phone Call		X					
CTRC Visit				X			
Online		X	X			X	X
**Participant Data**							
Consent		X					
Confirm Address		X					
Demographic			X				
Socio-economic			X				
KAP Survey			X				
**Clinical Data**							
Chart Review	X	X					
Health History			X				
Health Update				X		X	
Venous Blood*	X			X			
Remnant Samples	X						
**Environmental Data**							
Identify Exposure Area			X				
Tick Drags					X		
Outreach to Vet Clinics					X		
Geographic/Climate Data					X		

*Testing performed as part of routine care.

### Setting

North Carolina (NC) is the 9^th^ most populous state in the US and it has the largest census-defined rural population of any state besides Texas [16]. The state is generally classified into three distinct geographic regions, including the Blue Ridge Mountains to the west, the central Piedmont, and the Coastal Plains to the east. NC experiences some of the nation’s highest reported incidence rates of SFR and ehrlichiosis [7,17], most of which occur in the Piedmont region. In many years, NC accounts for more than 10% of SFR and 5% of ehrlichiosis cases reported nationally [7,17] Entomological studies demonstrate relatively high infection rates of rickettsiales among lone star and American dog ticks (*Dermacentor variabilis)* [18,19]. Along with the prevalence of bacteria found in ticks, seropositivity of SFGR and *Ehrlichia* are found in 20% and 10% of the general North Carolina population respectively [10].

### Screening & eligibility criteria

We leveraged the electronic health record (i.e., EPIC Systems, Verona, WI), to generate an automated, daily report of patients being tested for either SFR or ehrlichiosis. Each day, the report shows the medical record numbers (MRNs) of individuals who were tested for SFGR or ehrlichiosis testing through McLendon Clinical Laboratories. Study staff review the report and screen relevant clinical information from the medical record to determine eligibility for research purposes.

The eligibility criteria utilized a modified version of Council of State and Territorial Epidemiologists (CSTE) case definitions of Ehrlichiosis and SFG [20,21] (Table 2). Given the uncertainty of self-report of fever or home measurement of temperature, we did not require fever in our criteria. Notably, the 2023 CSTE case definition for ehrlichiosis – released after this project was launched - made similar changes [22].

**Table 2:** Eligibility criteria for patients tested for tick-borne illnesses in the UNC Health Care system.

Study inclusion criteria are the following:	Study exclusion criteria are the following:
Willingness to provide consent	Unable to access medical record from index visit (i.e., non-Epic user)
Adult (age ≥ 18 years)	Unwilling to provide consent
Resident of North Carolina	
Presents to frontline health provider (e.g., Primary Care, Urgent Care, or Emergency Department) with clinical symptoms suspicious for tick-borne disease, including at least two of the following:	
Fever – subjective or measured (temperature ≥ 38.0o C)	
Headache	
Rash or Eschar	
Arthralgia or Myalgia (aka “body aches”)	
Nausea, Vomiting, or Diarrhea	
Symptoms must be present for ≤ 14 days	
Diagnostic testing for SFGR and/or *Ehrlichia* ordered through McLendon Clinical Laboratories	

Select data are entered into a screening form in the electronic database (REDCap) [23]. Individuals not meeting the eligibility criteria are coded as ineligible for the enhanced surveillance project, but remnant samples are still included in the biobank. Those who meet the eligibility criteria are added to the eligible participants list to be contacted for participation.

### Recruitment and enrollment

Once the screening process is complete, eligible patients are added to a list for follow-up, and an initial email using the address documented in the medical record is sent. The email includes a brief description of the project objectives, a link to the webpage of the principal investigator (PI), and instructions on how to set up a call to discuss the study. All eligible patients are contacted by email through information available in the medical record. Patients are contacted up to three times by these same methods with at least two days between attempts. After three attempts and there is no reply, patients are marked as non-responders.

Once a patient responds, a phone call is scheduled between the patient and study staff. During the call, the staff member reviews both the goals of the surveillance project along with the objectives, methods, and risks/benefits of the research components with the patient. The patient is informed that all parts of the study are voluntary. The patients are given the opportunity to ask any questions. Questions that staff are unable to answer or those outside the scope of the study (e.g., concerns about clinical management) are referred to the PI.

Individuals who wish to participate are provided with an electronic version of the consent form (Supplementary Material) for digital signature via a secure platform. For individuals without access to the internet – or those preferring other communication mechanisms -- consent to participate is provided over the phone and documented by at least two team members. Once enrolled, each participant was assigned a unique study ID for linkage of data sources and samples.

### Enhanced surveillance activities

#### Baseline survey.

After consent is provided, the participant is added to the enrollment log and their contact information is added to the database. The participant is then emailed the baseline survey via REDCap [23] The baseline survey (Supplementary Material) elicits responses regarding demographic information (e.g., race, ethnicity, home address, insurance status, and employment) along with questions about tick and tick-borne disease knowledge, attitudes, and practices (e.g., prior exposures, personal protection, pets). The participant is given unlimited time to complete the survey and all questions are voluntary.

#### Convalescent testing.

If the patient agrees to collection of convalescent serum, - it is explained to the participant that this is consistent with the standard of care - staff place preliminary orders in EPIC, which are routed to the PI (RMB), a board-certified infectious diseases physician, for approval and signature. Once the tests are ordered, the patient is notified. The participant is able to go to any UNC Health facility with a laboratory to have phlebotomy performed at their convenience. The sera are subsequently sent to McLendon Clinical Laboratories for serological testing. When results are available in EPIC, staff add them to the REDCap database. The participants also receive their results within the patient web portal, if registered.

#### Household visit.

Participants are asked if they are willing to have an entomological survey performed at their home or presumed site of exposure. This is done in collaboration with the entomology team at North Carolina State University (NCSU). If the patient agrees, a field team surveys the household residence for ticks.If the participant was enrolled between the months of April and September, sampling would take place within two weeks of enrollment. If they enrolled outside of those months, sampling was scheduled for the following year. Other geographic factors are also recorded such as vegetation, size of yard, plant species, and type of home environment. If the participants have questions relating to tick collection or prevention, the collection team gives resources and advice for reducing tick exposure.

#### Research activities.

##### Recovery assessment

For the Recovery Assessment, follow-up surveys (Supplementary Material) are sent to participants approximately 30-, 60-, and 90-days after the initial clinical encounter. Participants confirm treatment completion and document other alternative diagnoses, along with the duration and nature of post-treatment symptoms. Symptoms of interest include domains assessing fatigue, pain, sleep, depression, activity, and perceptions of emotional and physical health.

###### Biorepository

During the initial review of the daily report, study staff utilizes the EHR to determine what remnant samples (e.g., whole blood, sera) are available after routine testing is completed. Specifically, whole blood from routine hematological or chemistry testing is collected within 48 hours, while remnant sera is collected after 90 days. Convalescent samples of participants completing the blood draw through the study are also collected routinely after 90 days.

### Data management

Study data will be collected and managed using REDCap electronic data capture tools hosted at [YOUR INSTITUTION].1,2 REDCap (Research Electronic Data Capture) is a secure, web-based software platform designed to support data capture for research studies, providing 1) an intuitive interface for validated data capture; 2) audit trails for tracking data manipulation and export procedures; 3) automated export procedures for seamless data downloads to common statistical packages; and 4) procedures for data integration and interoperability with external sources. Every day, the study staff receive new entries, which are entered into the database. Data quality checks have been built into the REDCap questionnaires (e.g., range of plausible values)

### Ethical considerations

Enhanced surveillance activities were determined to be within the existing standard care for clinical and public health practice and thus were determined to be exempt from human subjects research. However, all participants provided written agreement prior to any project activities. Additional elements including the convalescent surveys, abstraction of demographic and clinical information in association with remnant samples, and agreement to be contacted for future studies were approved by the UNC Institutional Review Board (21-0356).

## Results

Screening and remnant sample collection for enhanced surveillance started in August 2021. All available sera samples were collected and stored without regard for the eligibility criteria. As of April 2024, a total of 3,505 serum samples have been collected from 3,681 unique patients, including 2,739 acute samples and 671 paired acute and convalescent samples. After screening, a total of 1,062 (28.8%) met the eligibility criteria. The most frequent reasons for exclusion were less than two of the required symptoms (2089, 79.2%), symptoms lasting longer than 14 days (1630, 61.8%), and being under the age of 18 (278, 10.5%). The number of samples collected, number of eligible participants, and number of participants enrolled are shown in Table 3. Due to the COVID-19 pandemic, participant enrollment did not begin until July 2022 although remnant samples were collected and associated data was abstracted during this time. Since then, we have screened 3,133 patients of whom 770 were eligible, and subsequently enrolled 150. The majority of participants have completed all activities including surveys (140, 93.3%), convalescent blood draws (39, 26%), and household visits (55, 36.7%).

**Table 3:** Comparison of biorepository and enrollment data.

	All (n = 3681)		Eligible (n = 1062)		Enrolled (n = 150)	
Blood Sample	54	1.5%	19	1.8%	4	2.7%
Remnant Sera	3505		1017		148	
Acute	2739	78.1%	738	72.6%	45	30.4%
Convalescent	95	2.7%	26	2.6%	5	3.4%
Both	671	19.1%	253	24.9%	98	66.2%
Screening:						
≥18 Years of Age	3403	92.4%	1062	100%	150	100%
NC Resident	3568	96.9%	1062	100%	150	100%
Eligible Symptoms	1602	43.5%	1062	100%	150	100%
Eligible Duration	2060	56.0%	1062	100%	150	100%

**Fig 3:**
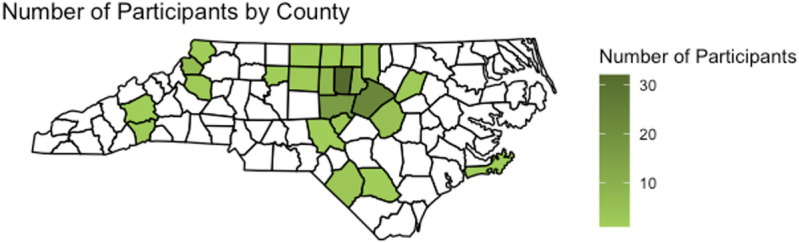
Map of Counties with Enrolled Participants. [14].

**Fig 4:**
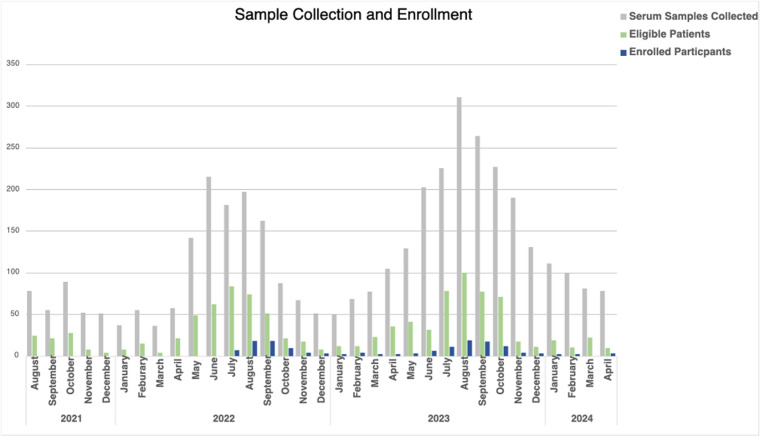
Sample Collection and Enrollment. Grey is the number of remnant samples collected from individuals tested for tick-borne disease. Green is the number of individuals eligible to participate in enhance surveillance program. Blue is the number of individuals enrolled by month.

Participants represent 26 of the 100 counties in NC (Fig 3) with the highest number from Orange, Wake, and Chatham counties, which are in close proximity to UNC Medical Center. Demographic characteristics of those tested, eligible, and enrolled are shown in Fig 4. Although we have screened over three thousand individuals, due to high volume of patients tested for TBDs, data entry has been completed for the dates between July 2021 to July 2023, which is 2285 individuals (Table 4). Notably, there was a female predominance (54.4% vs 45.3%) with the majority (1851, 81%) self-reporting as White/Non-Hispanic. For the enrolled cohort, (Table 5) most patients were seen in an outpatient clinic (70, 46.7%), but 21 (14%) were hospitalized. More than one-third (58, 38.7%) were aware of a tick bite at their visit, of which 70 (49.6%) suspect the tick exposure was the yard, which varied in size from <0.5 acres to > 10 acres. About 1-in-5 (28, 18.7%) reported travel around the time of the illness onset. Over 82% of patients tested for TBD were treated with antibiotics at the time of their visit, leaving 18% not treated with antibiotics. Patients were enrolled based on meeting eligibility criteria not on case diagnosis, therefore non-TBD cases were enrolled and were possibly treated for other infections.

**Table 4:** Demographic characteristics of individuals tested for tick-borne disease at UNC Health.

	All (n = 2285)		Eligible (n = 677)		Enrolled (n = 150)	
Age						
1-17	175	7.7%	0	0.0%	0	0.0%
18-28	269	11.8%	86	12.7%	12	8.0%
29-38	320	14.0%	125	18.5%	29	19.3%
39-48	334	14.6%	116	17.1%	33	22.0%
49-58	364	15.9%	115	17.0%	31	20.7%
59-68	391	17.1%	115	17.0%	20	13.3%
69-78	318	13.9%	91	13.4%	23	15.3%
78-88	105	4.6%	29	4.3%	2	1.3%
88-100	15	0.7%	0	0.0%	0	0.0%
Mean	48.3		50		49	
						
Sex						
Male	1036	45.3%	326	48.2%	66	44.0%
Female	1242	54.4%	349	51.6%	84	56.0%
Unknown	7	0.3%	1	0.1%	0	0.0%
						
Race						
White	1851	81.0%	558	82.4%	137	91.3%
Black or African American	209	9.1%	52	7.7%	8	5.3%
Asian	26	1.1%	12	1.8%	2	1.3%
American Indian or Alaskan	25	1.1%	4	0.6%	1	0.7%
Other Race	128	5.6%	36	5.3%	1	0.7%
Unknown	46	2.0%	13	1.9%	0	0.0%
						
Ethnicity						
Hispanic or Latino	107	4.7%	36	5.3%	2	1.3%
Not Hispanic or Latino	2136	93.5%	626	92.5%	147	98.0%
Unknown	44	1.9%	15	2.2%	1	0.7%

**Table 5:** Location, history of tick exposure, clinical symptoms, and laboratory abnormalities at the time of acute presentation for enrolled participants.

	Enrolled (n = 150)	
Screen Site		
Emergency Department	34	22.7%
Hospital	4	2.7%
Outpatient Clinic	70	46.7%
Urgent Care	35	23.3%
Other	4	2.7%
Unable to Determine	2	1.3%
		
Travel History	28	18.7%
		
Tick Bite	58	38.7%
		
Symptoms		
Fever	97	64.7%
Rash	63	42.0%
Eschar	8	5.3%
Headache	91	60.7%
Myalgia	92	61.3%
		
Lab Abnormalities		
Anemia	29	19.3%
Thrombocytopenia	19	12.7%
Hepatic Transaminase Elevation	31	20.7%
Leukopenia	15	10.0%
		
Hospitalization	21	14.0%
Death	0	0.0%
Antibiotics	124	82.7%

## Discussion

Herein, we describe the protocol for an enhanced surveillance project for tick-borne SFR and ehrlichiosis. Our early implementation results highlight some key findings. First, the low proportion of patients tested for TBD who were eligible for clinical symptoms inclusion (28.8%), was unexpected. Ideally, these tests would be ordered if TBD was suspected [8]. However, more than half of individuals tested either did not have at least two consistent symptoms or had experienced symptoms for more than 14 days; situations where the pre-test probability of SFGR or ehrlichiosis should be relatively low. This raises concerns regarding diagnostic stewardship, especially if testing is being performed in the context of chronic symptoms such as fatigue and arthralgia. Similarly, while fever is less prominent than previously thought, the absence of other typical symptoms suggests that many patients were being tested after potential tick exposure, even though patients were asymptomatic. Given that the majority of ticks are not infected with known human pathogens, testing after exposure, but in the absence of symptoms is not recommended [19,24].

It often is assumed most tick exposures take place when individuals are participating in outdoor occupational or recreational activities. However, nearly half of our participants (49.5%) reported that they were likely exposed in or around their home. A somewhat smaller proportion (43.2%) reported that the exposure likely occurred in relation to work or travel. With the number of tick exposures increasing, even at the patient’s home, research on control of ticks in the peridomestic space, especially lone star ticks, which transmit *Ehrlichia* spp. and induces the mammalian meat allergy, alpha-gal syndrome, is urgently needed. With increasing exposures believed to be at the home, tick control education is also valuable for awareness and tick bite prevention.

Our project demonstrates the value of integrating active, automated EHR systems for disease surveillance. For example, from the 150 enrolled participants in our cohort we reported 15 and 20 confirmed cases of SFR and ehrlichiosis, respectively. In contrast, the North Carolina State Health Department reported only 14 and 11 confirmed cases of SFR and ehrlichiosis respectively, in 2022, which accounts for the entire state [25,26]. However, this could be due to the changes in case definition for ehrlichiosis in 2020 and 2022. The main change in the definition is that fever is now not one of the required symptoms to define a case. Fever is still required for the case definition of SFR. These results show how the enhanced surveillance approach can provide more and higher quality epidemiological data. Lastly, our biorepository, which we intend to make available to other researchers, opens the doors for future investigation. With over four thousand samples collected to date, further analysis can be performed as well as the opportunity for the validation of new testing methods.

The approach, however, also has some limitations. Foremost among these is the potential for selection bias. Specifically, individuals with a known tick exposure or positive initial titer result may be more likely to participate than those without. While the project seeks to enroll individuals with high probability of TBD, many patients with these infections do not find ticks and may have a negative initial test. Therefore, it is possible that such individuals are underrepresented in our cohort. We are reassured that the demographic characteristics of the enrolled individuals appear broadly similar to those of the larger pool of tested individuals, but this will require further review as the project continues. We also acknowledge that patients under 18 are also at risk for tick-borne illness and were excluded from this study. Due to extra criteria required by UNC IRB, we decided to limit the study to those at consenting age, but this could be something further explored in future studies. Furthermore, testing and survey collection dates do not always take place on exact dates. We attempt to ensure that participants can complete the tasks appropriately, but since there is not a required due date, some participants may complete the testing or surveys at a later date, which can affect direct comparisons. For most investigations, however, minor variations (i.e., within 7–10 days) are not expected to adversely impact our conclusions.

## Conclusion

Tick-borne diseases are on the rise in the United States. The enhanced surveillance project described herein attempts to address some of these key knowledge gaps through the use of detailed, online case report forms and incentivized obtainment of convalescent samples for testing. In addition, we use the epidemiology of TBDs to guide the conduct of entomological surveys, thereby yielding the most relevant assessment of the individual’s risk of exposure. Results of the study will inform future investigations and potentially lead to intervention and prevention methods in the state of North Carolina when it comes to tick-borne illnesses.

## Supporting Information

S1 File First Contact Email. (PDF)

S2 File Consent Form. (PDF)

S3 File HIPAA Form. (PDF)

S4 File Screening Form. (PDF)

S5 File Acute Visit Summary. (PDF)

S6 File Labs and Imaging. (PDF)

S7 File Baseline Survey. (PDF)

S8 File 30Day Follow Up Survey. (PDF)

S9 File 60Day Follow Up Survey. (PDF)

S10 File 90Day Follow Up Survey. (PDF)
