# Supplementary material for: Enhanced surveillance for tick-borne rickettsiosis and ehrlichiosis in North Carolina: Protocol and preliminary results
Source: PLoS One. 2025 May 12;20(5):e0320361. doi: 10.1371/journal.pone.0320361 (PMC12068726; doi:10.1371/journal.pone.0320361)
Supplement: S1 File — (PDF) [file pone.0320361.s001.pdf]

First Contact Email To Eligible Participants:

Dear Future Participant,

My name is XXX I am the study coordinator for the Tick-Borne Disease in Central North Carolina Study (TBD-NC) being led by Dr. Ross Boyce at the University of North Carolina at Chapel Hill. We are reaching out to you because you were recently tested for tick-borne disease and may be eligible to participate in our research study. The purpose of this study is to learn more about how many people are infected by ticks and what symptoms they experience. The results may help guide prevention and control strategies.

As a next step, I would like to schedule a phone call to speak with you about the study. During the call, I will share information about the study objectives, methods, and risks/benefits. You will also have time to ask us any questions you might have about the study. If you agree to participate, I will send you the consent forms through a secure email link.

If you could provide a phone number and good days/times to schedule the call that would be great.

Thank you and I look forward to speaking with you!
