# Supplementary material for: Enhanced surveillance for tick-borne rickettsiosis and ehrlichiosis in North Carolina: Protocol and preliminary results
Source: PLoS One. 2025 May 12;20(5):e0320361. doi: 10.1371/journal.pone.0320361 (PMC12068726; doi:10.1371/journal.pone.0320361)
Supplement: S2 File — (PDF) [file pone.0320361.s002.pdf]

University of North Carolina at Chapel Hill
Consent to Participate in a Research Study
Adult Enrollment and Specimen Consent

Consent Form Version Date: v1.0 dated September 14, 2021
IRB Study # 21-0356
Title of Study: IGHID 12102 - Tick-Borne Disease in Central North Carolina Study (TBD-NC)
Principal Investigator: Ross M Boyce
Principal Investigator Department: Medicine-Infectious Diseases
Principal Investigator Phone number: 919-966-2537
Principal Investigator Email Address: ross_boyce@med.unc.edu

Funding Source and/or Sponsor: Creativity Hub Award (Office of Vice Chancellor for Research)
IDSA GERM Award

Study Contact Email: unc_tick_study@med.unc.edu

CONCISE SUMMARY

The purpose of this study is to learn more about tick-borne diseases such as Rocky Mountain Spotted Fever and Erlichiosis in North Carolina. Specifically, we are trying to determine how many people are infected each year and what symptoms they experience. In addition, we are seeking to identify individual, household, and geographic risk factors that may place individuals at higher risk of tick-borne infections.

The expected duration of participation for each individual is approximately 3 months At your standard of care follow up visit, a venous blood draw will be performed. For the purpose of this study, we will collect one additional container of blood (an additional of not more than 20 mL of blood will be collected). Basic information about you, your health history, and your symptoms will be collected through a series of questionnaires that you can complete online. We will also ask your permission to have a team from North Carolina State University visit your home or other location where you think you were bitten to look for ticks. If a pet dog is present in the home, we will also ask your permission to access records of any tick-borne disease testing done for the pet at a veterinary clinic.

Risks to being part of this study include: 1) risks associated with blood draws include bruising, mild pain, or discomfort from blood sampling that goes away soon and in very rare cases infection, might occur; 2) risk of breach of confidentiality and 3) risk of exposure to SARS-CoV-2 related to leaving home for a study visit. We will be very careful to try to minimize these risks. You will benefit by learning more detailed information about your potential infection, which is not routinely performed, as well as potential risks around your home, which should help you prevent future infections. At a societal level, the information gathered from this study will help public health officials and the scientific community understand the burden of tick-borne illness in central North Carolina and develop new strategies to protect residents.

What are some general things you should know about research studies?
You are being asked to take part in a research study. Joining the study is voluntary.
You may choose not to participate, or you may withdraw your consent to be in the study, for any reason, without penalty.

Research studies are designed to obtain new knowledge. This new information may help people in the future. You may not receive any direct benefit from being in the research study. There also may be risks to being in research studies.

Deciding not to be in the study or leaving the study before it is done will not affect your relationship with the researcher, your health care provider, or the University of North Carolina-Chapel Hill. If you are a patient with an illness, you do not have to be in the research study in order to receive health care.

Details about this study are discussed below. It is important that you understand this information so that you can make an informed choice about being in this research study.

You will be given a copy of this consent form. You should ask the researchers named above, or staff members who may assist them, any questions you have about this study at any time.

What is the purpose of this study?
The purpose of this research study is to generate more accurate estimates of tick-borne disease incidence in central North Carolina. We would like to better understand the demographic, socio-economic, behavioral and geographic risk factors for tick-borne disease infection among residents of central North Carolina. We would also like to describe the range and severity of symptoms and characteristics of tick-borne infections.

Are there any reasons you should not be in this study?
You should not be in this study if you are unwilling to allow the collection of samples required by this study or do not wish to complete the surveys that we will send to you.

How many people will take part in this study?
Approximately 500 people will take part in this study.

How long will your part in this study last?
Your participation in this study will last about 3 months.

What will happen if you take part in the study?
If you decide to participate in this study, we will first conduct an electronic survey to gather information on your demographic and socioeconomic information, health history related to tick-borne infections, knowledge of local tick-borne disease risk, and pictures of any skin rashes you may have.

you will visit the Clinical Trials Research Center at UNC to measure basic vital signs like height, weight, blood pressure, and heart rate. You will also complete a health update questionnaire and a standard of care venous blood draw will be performed, with one additional container of blood collected (an additional of not more than 20 mL of blood will be collected.

Approximately 60 and 90 days after your enrollment, you will complete additional online surveys regarding your treatment, other diagnoses, and continued symptoms.

Finally, if you have a pet dog in your home, we will ask you to sign a consent form to release your dog's veterinary records with information on tick-borne disease testing that is frequently done at annual physical exams.

What tests are being performed on the blood samples collected in this study, and when will you receive results?

Test	Purpose of test	FDA-approval status?	Results returned to the participant?	Timing of result return
Rocky Mountain Spotted Fever Immunofluorescence antibody (Biocell Diagnostics)	Measure your antibody levels again *Rickettsia* bacteria	FDA-approved	Yes	Approximately 1-2 weeks after the CTRC visit
Ehrlichia IgG (Biocell Diagnostics)	Measure your antibody levels again *Ehrlichia* bacteria	FDA-approved	Yes	Approximately 1-2 weeks after the CTRC visit
Rocky Mountain Spotted Fever PCR	Detects the DNA of Rickettsia bacteria in your blood	FDA-approved	Yes, but will only be performed if enough blood leftover from your first, non-study visit	N/A
Ehrlichia PCR	Detects the DNA of Ehrlichia bacteria in your blood	Research use only (Not FDA-approved)	No	N/A
Rickettsia and Erlichia species specific IFA (Fuller Laboratories)	Identifies the specific species of Rickettsia or Ehrlichia	Research use only (Not FDA-approved)	No	N/A
C-reactive protein (Medix Biochemica)	Measures amount of inflammation	Research use only (Not FDA-approved)	No	N/A

What are the possible benefits from being in this study?
Less than 3% of all patients in the state currently receive confirmation of their diagnosis because many medical providers do not perform both acute and convalescent testing as recommended by the CDC. Furthermore, many patients do not get testing for both Rickettsia and Ehrlichia because many providers are not aware that Ehrlichia is a frequent cause of tick-borne illness. Full participation in the study will ensure that you receive both tests. In addition, we may be able to provide you with additional information about the presence of ticks around your house. You will also be contributing to the creation of knowledge that may benefit others in society.

What are the possible risks or discomforts involved from being in this study?
Blood Collection: Drawing blood may cause some discomfort, bruising, or bleeding. You may experience discomfort from the insertion of the needle in your vein. Rarely, people may faint as a result of drawing blood. Blood will be drawn by phlebotomist. This staff is experienced in blood drawing and prepared to handle any discomfort, bruising, bleeding or fainting you may experience.

Breach of Confidentiality: It is possible that your protected health information may become known to persons not directly involved in the conduct of this study without our knowledge or intention. The study investigators will do their best to make sure that this does not happen by storing all information on computers that are protected by security passwords, and by ensuring any paper copies of information are placed in locked file cabinets.

Disclosure of Personal Information: There is a small risk of disclosure of household location, but it is very unlikely that this would adversely impact participants as there is not significant stigma risk of social isolation associated with tick-borne illness.

SARS-CoV-2 (Covid-19) Exposure: There is a risk of exposure to SARS-CoV-2 infection associated with leaving the home for study visits. This risk will likely fluctuate with changes in disease transmission and local policies. We will minimize this risk by following standard precautions such as physical distancing, mask wearing, and hand washing.

What if we learn about new findings or information during the study?
You will be given any new information gained during the course of the study that might affect your willingness to continue your participation.

How will information about you be protected?
Participants will not be identified in any report or publication about this study. We may use de-identified data and/or specimens from this study in future research without additional consent. Although every effort will be made to keep research records private, there may be times when federal or state law requires the disclosure of such records, including personal information. This is very unlikely, but if disclosure is ever required, UNC-Chapel Hill will take steps allowable by law to protect the privacy of personal information. In some cases, your information in this research study could be reviewed by representatives of the University, research sponsors, or government agencies (for example, the FDA) for purposes such as quality control or safety.

Will my genetic information be shared?
Your blood and tissue samples contain genes that are made of DNA unique to you. To do more powerful research, it is helpful for researchers to share information they get from studying human samples. They do this by putting it into one or more scientific databases, where it is stored along with information from other studies. Researchers can then study the combined information to learn even more about health and disease. If you agree to take part in this study, some of your genetic and health information might be placed into one or more scientific databases. There are many different kinds of scientific databases; some are maintained by this institution, some are maintained by the federal government, and some are maintained by private companies. For example, the National Institutes of Health (an agency of the federal government) maintains a database called "dbGaP." A researcher who wants to study the information must apply to the database. Different databases may have different ways of reviewing such requests. Researchers with an approved study may be able to see and use your information, along with information from many other people. Your name and other information that could directly identify you (such as address or social security number) will never be placed into a scientific database. However, because your genetic information is unique to you, there is a small chance that someone could trace it back to you. The risk of this happening is very small, but may

grow in the future as technology advances. Researchers will always have a duty to protect your privacy and to keep your information confidential.

Federal law called the Genetic Information Nondiscrimination Act (GINA) generally makes it illegal for health insurance companies, group health plans, and most employers to discriminate against you based on your genetic information. GINA does not protect you against genetic discrimination by companies that sell life insurance, disability insurance, or long-term care insurance. GINA also does not protect you against discrimination based on an already-diagnosed genetic condition or disease.

By signing this informed consent document, you agree that some of the information generated by participating in this study and/or a copy of the consent form may be included in your medical record and that this information may be viewed by other physicians or caregivers who provide healthcare services to you. This will allow the doctors caring for you to know what study medications or tests you may be receiving as a part of the study and know how to take care of you if you have other health problems or needs during the study. Additionally, the information may be shared with your medical insurance plan if the research services provided are billed to your insurance.

Under North Carolina law, confidentiality does not extend to certain communicable diseases, such as TB, HIV, hepatitis, or other illnesses that put others at risk. If the researchers become aware that subjects have such an illness, they are required to report it to state authorities.

<u>Will you receive results from research involving your specimens?</u>
Most research with your specimens is not expected to yield new information that would be meaningful to share with you personally. There are no plans to re-contact you or other subjects with information about research results. The use of your samples will not result in commercial profit. You will not be compensated for the use of your samples other than what is described in this consent form. You will be asked to sign a separate form ("HIPAA Authorization") to allow researchers to review your medical records.

<u>What will happen to the specimens?</u>
Any leftover sample after completion of all study required testing will be stored in the Boyce Lab at UNC for future research studies. This may include studies examining genetic information about the virus or your body's response, including whole genome sequencing. Future research may include pathogen genetic testing and testing of your genes or your DNA (your own genetic information). We do not know whether a type of testing called whole genome sequencing, or WGS, might be done. In WGS, researchers look at all of your genes and at almost all of your DNA. Researchers may want to share genetic information (with protection of your identity) with other researchers around the world, so that they can learn more about the causes and treatment of diseases. They may store this information in dbGaP, a genetic database maintained by the National Institutes of Health, as well as in other protected databases

<u>Who owns the specimens?</u>
Any blood, body fluids, or tissue specimens obtained for this purpose become the exclusive property of the University of North Carolina at Chapel Hill. This organization may retain, preserve or dispose of these specimens and may use these specimens for research that may result in commercial

applications. There are no plans to compensate you for any future commercial use of these specimens.

Can you withdraw the specimens?

You may withdraw your specimen in the future if you change your mind. You should contact the researchers on the front page of this form. It is best to make your request in writing.

Any analysis in progress at the time of your request or already performed prior to your request being received by the researcher will continue to be used as part of the research study. Once the researchers have been notified, your remaining specimens would be destroyed. If you do not make such a request, the specimens may be stored forever. The researchers may choose to destroy the specimens at any time.

What will happen if you are injured by this research?

All research involves a chance that something bad might happen to you. If you are hurt, become sick, or develop a reaction from something that was done as part of this study, the researcher will help you get medical care, but the University of North Carolina at Chapel Hill has not set aside funds to pay you for any such injuries, illnesses or reactions, or for the related medical care. Any costs for medical expenses will be billed to you or your insurance company. You may be responsible for any co-payments and your insurance may not cover the costs of study related injuries.

If you think you have been injured from taking part in this study, call the Principal Investigator at the phone number provided on this consent form. They will let you know what you should do.
By signing this form, you do not give up your right to seek payment or other rights if you are harmed as a result of being in this study.

What if you want to stop before your part in the study is complete?

You can withdraw from this study at any time, without penalty. The investigators also have the right to stop your participation at any time. This could be because you have had an unexpected reaction, or have failed to follow instructions, or because the entire study has been stopped.

If you withdraw or are withdrawn from this study all data collected up until the point of withdrawal will be retained, however no additional information will be collected unless you provide additional written permission for further data collection at the time of your withdrawal.

Will you receive anything for being in this study?

You will receive $5 for each survey completed.

Will it cost you anything to be in this study?

We are using a FDA-approved test performed in a certified laboratory (UNC McLendon Clinical Laboratories) so that we can return the results to you. Your insurance may be billed for these costs, but the testing is considered standard of care for these diseases. Therefore, your insurance may charge you a copay or other costs similar to what you would be charged for laboratory testing performed by your doctor.

Who is sponsoring this study?
This research is funded by the Centers for Disease Control and Prevention (CDC) Epidemiology and Laboratory Capacity Funds, via the North Carolina Department of Health and Human Services. This means that the research team is being paid by the sponsor for doing the study. The researchers do not, however, have a direct financial interest with the sponsor or in the final results of the study.

What if you have questions about this study?
You have the right to ask, and have answered, any questions you may have about this research. If you have questions about the study (including payments), complaints, concerns, or if a research-related injury occurs, you should contact the researchers listed on the first page of this form.

What if you have questions about your rights as a research participant?
All research on human volunteers is reviewed by a committee that works to protect your rights and welfare. If you have questions or concerns about your rights as a research subject, or if you would like to obtain information or offer input, you may contact the Institutional Review Board at 919-966-3113 or by email to IRB_subjects@unc.edu.

Participant's Agreement:

I have read the information provided above. I have asked all the questions I have at this time. I voluntarily agree to participate in this research study.

Tests performed on your blood and samples may indicate that you may qualify for additional research studies. Please indicate if we may contact you for other future research studies by writing your initials below.

Yes ☐ No ☐ I agree to be contacted for future research studies.

Please write your initials in the appropriate space below if you agree to allow genetic testing to be done on your stored specimen(s) in the future.

Yes ☐ No ☐ I agree to allow genetic testing to be done on my stored specimen(s) in the future.

Signature of Research Participant	Date
Printed Name of Research Participant	
Signature of Research Team Member Obtaining Consent	Date
Printed Name of Research Team Member Obtaining Consent	
Signature of Witness if applicable; e.g. literacy issues, visually impaired, physically unable to sign, witness/interpreter for non-English speaking participants using the short form)	Date
Printed Name of Witness	
