# Supplementary material for: Enhanced surveillance for tick-borne rickettsiosis and ehrlichiosis in North Carolina: Protocol and preliminary results
Source: PLoS One. 2025 May 12;20(5):e0320361. doi: 10.1371/journal.pone.0320361 (PMC12068726; doi:10.1371/journal.pone.0320361)
Supplement: S4 File — (PDF) [file pone.0320361.s004.pdf]

Screening Form

Please complete the survey below.Thank you!

Epic Medical Record Number	______________________
Date of Screening	______________________
Screener	◯<br>◯<br>◯<br>◯<br>◯<br>(Enter Name)
If other, please enter full name	______________________
Did participant consent to study?	◯ Yes<br>◯ No
Was any blood sample available? (CORRECTED)	◯ No<br>◯ Yes
Was the sample from an acute or convalescent visit?	◯ Acute<br>◯ Convalescent
Check all factors that explain why no blood sample was available at this time.	☐ Participant did not present in report throughout participation<br>☐ No labs ordered by provider requiring blood draw<br>☐ Sample pulled from Core lab other group/lab<br>☐ Report error or delay (ex. not loading information within the necessary window)
If through other lab, which UNC lab?	______________________
Was remnant sera available?	◯ Yes<br>◯ No
Acute or Convalescent?	◯ Acute<br>◯ Convalescent<br>◯ Both<br>◯ 3+ samples

Eligibility Criteria

Age ≥ 18 years	◯ Yes<br>◯ No
Resident of North Carolina?	◯ Yes<br>◯ No
Does patient report at least two of the following symptoms?<br><br>- Fever<br>- Headache<br>- Rash or Eschar (scab)<br>- Arthralgia (joint pains) or Myalgia (body aches)<br>- Nausea, Vomiting, or Diarrhea	◯ Yes<br>◯ No
Did patient meet clinical symptoms eligibility requirements?	◯ Yes<br>◯ No
Symptoms present for less than 14 days?	◯ Yes<br>◯ No
ELIGIBLE	
NOT ELIGIBLE	
Screener Confirmation	◯ Not Eligible<br>◯ Eligible
