# Supplementary material for: Enhanced surveillance for tick-borne rickettsiosis and ehrlichiosis in North Carolina: Protocol and preliminary results
Source: PLoS One. 2025 May 12;20(5):e0320361. doi: 10.1371/journal.pone.0320361 (PMC12068726; doi:10.1371/journal.pone.0320361)
Supplement: S5 File — (PDF) [file pone.0320361.s005.pdf]

Acute Visit Summary

Study ID	______________________
Patient FIRST Name:	______________________
Patient LAST Name:	______________________
Street Address:	______________________
City:	______________________
Zip Code:	______________________
County of Residence:	______________________
Patient Sex:	◯ Male<br>◯ Female<br>◯ Unknown
Patient Date of Birth:	______________________
Race:	◯ White<br>◯ Black or African American<br>◯ American Indian or Alaska Native<br>◯ Asian<br>◯ Native Hawaiian or Other Pacific Islander<br>◯ Other Race<br>◯ Unknown<br>◯ Refused
Ethnicity:	◯ Hispanic or Latino<br>◯ Not Hispanic or Latino<br>◯ Patient Refused<br>◯ Unknown
Date of Initial Visit	______________________
At what type of health facility was the patient seen?	◯ Outpatient Clinic<br>◯ Urgent Care<br>◯ Emergency Department<br>◯ Hospital<br>◯ Other<br>◯ Unable to determine

If seen at other type of facility, please list here: __________________________________

What was the name of the clinic, emergency department, or hospital where the patient was seen? __________________________________

What was the level of training of the primary healthcare provider
- ◯ Attending Physician (MD, DO)
- ◯ Resident Physician
- ◯ Physician Assistant
- ◯ Nurse Practitioner
- ◯ Unable to determine

Exposure History

In the two weeks before symptom onset or diagnosis (use earlier date), did the patient travel out of their county, state, or country of residence?
- ◯ Yes
- ◯ No
- ◯ Unknown

Destination (county, state or country): ____________________________

When did they arrive? ____________________________

When did they depart? ____________________________

In the two weeks before symptom onset or diagnosis (use earlier date), did the patient notice any tick bites?
- ◯ Yes
- ◯ No
- ◯ Unknown

If yes, date: ____________________________

If the patient removed a tick from their body, what was the geographic location at the time (county, state, or country)? __________________________________

Clinical evidence of tickborne rickettsial disease:

	Yes	No	Unknown
Fever (Temp ≥38 C)	◯	◯	◯
Rash	◯	◯	◯
Eschar	◯	◯	◯
Headache	◯	◯	◯
Myalgia	◯	◯	◯
Anemia (Hemoglobin < 13.5 g/dL for men or < 12 g/dL for women)	◯	◯	◯

Thrombocytopenia (Platelets < 150)	◯	◯	◯
Hepatic transaminase elevation (AST or ALT > 45)	◯	◯	◯
Leukopenia (WBC < 4.0)	◯	◯	◯
Other	◯	◯	◯

Other, specify: __________________________________

Date of illness onset: ____________________________

Did the patient experience any severe complications in the clinical course of this illness?
◯ Yes
◯ No
◯ Unknown

If the patient experienced severe complications due to this illness, specify the complication(s):
☐ Acute respiratory distress syndrome (ARDS)
☐ Disseminated intravascular coagulation (DIC)
☐ Meningitis/encephalitis
☐ Organ failure
☐ Other

Other, specify: __________________________________

At the time of diagnosis, was the patient immunocompromised due to medical condition(s) or treatment(s) (such as one of the following: chemotherapy for current illness, HIV, anti-rejection drugs post-transplant, corticosteroids >14 days [such as prednisone, methylprednisolone, or dexamethasone], rheumatoid arthritis [with use of immunomodulator])?
◯ Yes
◯ No
◯ Unknown

Specify condition(s) or treatment(s): __________________________________

Was the patient hospitalized because of this illness?
◯ Yes
◯ No
◯ Unknown

Admission date: ____________________________

Discharge date: ____________________________

Did the patient die from this illness or complications of this illness?
◯ Yes
◯ No
◯ Unknown

If yes, date: ____________________________

Were antibiotics prescribed for this infection?	◯ Yes<br>◯ No<br>◯ Unknown
Specify antibiotic(s):	☐ Doxycycline<br>☐ Amoxicillin<br>☐ Azythromycin<br>☐ Other
Other, specify:	______________________
Date treatment was prescribed:	______________________
Prescribed dose<br><br>Document both dose and frequency (e.g., 100 mg twice daily)	______________________
Prescribed duration (days):	______________________
In the year before symptom onset or diagnosis (use earlier date), did the patient receive a blood transfusion?	◯ Yes<br>◯ No<br>◯ Unknown
Date of blood transfusion:	______________________
What type of blood product?	☐ PRBC<br>☐ Platelets<br>☐ FFP
Was the patient's infection transfusion-associated?	◯ Yes<br>◯ No<br>◯ Unknown
In the year before symptom onset or diagnosis (use earlier date), did the patient receive an organ transplant?	◯ Yes<br>◯ No<br>◯ Unknown
Date of transplant:	______________________
Was the patient's infection transplant-associated?	◯ Yes<br>◯ No<br>◯ Unknown
If the patient received an organ transplant, specify which organ(s):	______________________
