# Supplementary material for: Enhanced surveillance for tick-borne rickettsiosis and ehrlichiosis in North Carolina: Protocol and preliminary results
Source: PLoS One. 2025 May 12;20(5):e0320361. doi: 10.1371/journal.pone.0320361 (PMC12068726; doi:10.1371/journal.pone.0320361)
Supplement: S6 File — (PDF) [file pone.0320361.s006.pdf]

Labs and Imaging

Please complete the survey below.Thank you!

Vital Signs

Systolic Blood Pressure

____
(mm Hg)

Diastolic Blood Pressure

____
(mm Hg)

Heart Rate / Pulse

____
(beats per min)

Oxygenation (SpO2)

Usually listed as a percent

____

Weight

____
(kg)

Initial Laboratory Studies (Routine)

Only enter test results as ordered by clinical provider

Date Labs Drawn

____

Sodium

____
(mmol/L)

Bicarbonate (CO2)

____
(mmol/L)

BUN

____
(mg/dL)

Creatinine

____
(mg/dL)

WBC

____
(x 10^9/L)

Absolute Lymphocyte Count

____
(x 10^9/L)

Hemoglobin

__________________ (g/dL)

AST

__________________ (units/L)

ALT

__________________ (units/L)

Total Bilirubin

__________________ (mg/dL)

Platelet Count

__________________ (x 10^9/L)

Lactate

__________________ (mmol/L)

C-reactive protein (CRP)

__________________ (mg/L)

Erythrocyte Sedimentation Rate (ESR)

__________________ (mm/hr)

Serological Testing for Rickettsia

Was serological testing for SFGR/RMSF ordered at initial visit (e.g. acute sample)?

◯ Yes
◯ No

If not ordered, was a remnant sample available for testing

◯ Yes
◯ No

Acute SFGR/RMSF IgG result?

◯ Negative
◯ Positive (≥1:64)

Acute titer result, if positive

◯ 1:64
◯ 1:128
◯ 1:256
◯ 1:512
◯ 1:1024
◯ ≥1:2048

Convalescent serology collection date?

____

Convalescent SFGR/RMSF IgG result?

◯ Negative
◯ Positive (≥1:64)

Convalescent titer result, if positive	◯ 1:64<br>◯ 1:128<br>◯ 1:256<br>◯ 1:512<br>◯ 1:1024<br>◯ ≥1:2048
Was there a fourfold change in antibody titer between the two IgG serum specimens?	◯ Yes<br>◯ No

Serological Testing for Ehrlichia

Was serological testing for Ehrlichia ordered at initial visit (e.g. acute sample)?	◯ Yes<br>◯ No
If not ordered, was a remnant sample available for testing	◯ Yes<br>◯ No
Acute Ehrlichia IgG result?	◯ Negative<br>◯ Positive (≥1:64)
Acute titer result, if positive	◯ 1:64<br>◯ 1:128<br>◯ 1:256<br>◯ 1:512<br>◯ 1:1024<br>◯ ≥1:2048
Convalescent serology collection date?	________________________
Convalescent Ehrlichia IgG result?	◯ Negative<br>◯ Positive (≥1:64)
Convalescent titer result, if positive	◯ 1:64<br>◯ 1:128<br>◯ 1:256<br>◯ 1:512<br>◯ 1:1024<br>◯ ≥1:2048
Was there a fourfold change in antibody titer between the two IgG serum specimens?	◯ Yes<br>◯ No

Serological Testing for Lyme

Was serological testing for Lyme disease ordered at initial visit?<br><br>Note: Current Lyme diagnostic algorithms do not recommend testing of acute and convalescent samples	◯ Yes<br>◯ No
Lyme Serology result?	◯ Negative (≤0.90)<br>◯ Equivocal (0.91 - 1.09)<br>◯ Positive (≥1.09)

Lyme Western Blot Result

Positive defined as

- IgM Criteria - At least 2 of 3 IgM bands present
- IgG Criteria - At least 5 of 10 IgG bands present

◯ IgM (-) / IgG (-)
◯ IgM (+) / IgG (-)
◯ IgM (-) / IgG (+)
◯ IgM (+) / IgG (+)
(Select one)

Other Testing Ordered at Initial Visit

Other Diagnostic Tests:

☐ PCR for Rickettsia (sent to State Lab)
☐ PCR for Ehrlichia (sent to Mayo)
☐ Morulae visualization
☐ PCR UNC McLendon
☐ Other
(Check all that apply)

Rickettsia PCR source

◯ Blood or Serum
◯ Skin swab or biopsy
◯ Other

Rickettsia PCR result

◯ Positive
◯ Negative

If Rickettsia PCR, immunostain, or sequencing performed and positive, specify genus or species identified:

◯ Rickettsia africae
◯ Rickettsia parkeri
◯ Rickettsia rickettsii
◯ Rickettsia species 364D
◯ Rickettsia species (pan-Rickettsia)
◯ Spotted fever group Rickettsiae
◯ Other, specify:

Other, specify:

____

Ehrlichia PCR source

◯ Blood or Serum
◯ Skin swab or biopsy
◯ Other

Ehrlichia PCR result

◯ Negative
◯ Positive

If Ehrlichia PCR, immunostain, or sequencing performed and positive, specify genus or species identified:

◯ Ehrlichia chaffeensis
◯ Ehrlichia ewingii
◯ Ehrlichia muris eauclairensis
◯ Ehrlichia species (pan-Ehrlichia)
◯ Genera Ehrlichia/Anaplasma
◯ Other, specify:

UNC McLendon Ehrlichia PCR result

◯ Negative
◯ Positive

Other, specify:

____

Other testing or general comments

____

Case Definition

Case Definition	◯ Confirmed<br>◯ Probable<br>◯ Suspect<br>◯ Not a Case<br>◯ Unknown
Comments:	____
