# Supplementary material for: Enhanced surveillance for tick-borne rickettsiosis and ehrlichiosis in North Carolina: Protocol and preliminary results
Source: PLoS One. 2025 May 12;20(5):e0320361. doi: 10.1371/journal.pone.0320361 (PMC12068726; doi:10.1371/journal.pone.0320361)
Supplement: S7 File — (PDF) [file pone.0320361.s007.pdf]

Baseline: Demographics, KAP, Exposure History

Please complete the survey below.Thank you!

Languages:	◯ English<br>◯ Español
Survey date	__________________
First Name:	__________________
Last Name:	__________________
What is your date of birth?	__________________
What is your age?	__________________
County of residence:	__________________
What is your highest education level?	◯ High school or lower<br>◯ Some college<br>◯ Bachelors degree<br>◯ Graduate or Professional degree
Sex:	◯ Male<br>◯ Female
How tall are you? (Feet)	__________________<br>(feet)
How tall are you? (Inches)	__________________<br>(inches)
How much do you weigh (lbs)?	__________________<br>(pounds)
What type of health insurance do you have?	☐ Private health insurance<br>☐ Medicare<br>☐ Medicaid<br>☐ None<br>☐ Other<br>(select all that apply)
If you have another type of insurance, please specify:	______________________

EMPLOYMENT/WORK

Are you currently employed in any capacity?

- ◯ Yes
- ◯ No

Occupation (please be as specific as possible): __________________________________

Who is your employer? __________________________________

What is your salary?

- ◯ More than $150,000
- ◯ $100,000 - $149,999
- ◯ $50,000 - $99,999
- ◯ Less than $50,000

Do you frequently work outdoors?

- ◯ Yes
- ◯ No

Please describe the kind of work you do: ____

Knowledge, Attitudes, and Practices Related to Tick-Borne Diseases

How would you rate your knowledge of tick-borne diseases?

- ◯ A lot
- ◯ Some
- ◯ A little
- ◯ None

Which of these tick-borne diseases do you think are found in your area?

- ☐ None
- ☐ Anaplasmosis
- ☐ Babesiosis
- ☐ Bourbon Virus
- ☐ Ehrlichiosis
- ☐ Heartland Virus
- ☐ Lyme disease
- ☐ Powassan Virus
- ☐ Rocky Mountain Spotted Fever
- ☐ Southern Tick Associated Rash Illness (STARI)
- ☐ Severe Fever with Thrombocytopenia Syndrome
- ☐ Tularemia
- ☐ Do not know

(select all that apply)

How would you describe the prevalence of tick-borne diseases in your area?

- ◯ Common
- ◯ Very Common
- ◯ Somewhat Common
- ◯ Rare

What kind of ticks are found in your area?

- ☐ None
- ☐ Deer Ticks (also known as Black Legged Ticks)
- ☐ Dog Ticks
- ☐ Gulf Coast Ticks
- ☐ Lone Star Ticks
- ☐ Long-Horned Ticks
- ☐ Do not know

(select all that apply)

Where do you think you can find ticks?	☐ Dogs<br>☐ Dirt<br>☐ Other<br>☐ Do not know
Other, please specify:	__________________________
How would you describe the health consequences of being infected with a tick-borne disease?	◯ Very Severe<br>◯ Somewhat Severe<br>◯ Not Severe / Mild
What do you think is the likelihood of contracting a tick-borne disease in the next year?	◯ Very Likely<br>◯ Moderately Likely<br>◯ Not Likely
Have you ever been diagnosed with a tick-borne disease?	◯ Yes<br>◯ No
Which one(s)?	☐ Rickettsial Disease (e.g., Rocky Mountain Spotted Fever)<br>☐ Ehrlichiosis<br>☐ Lyme Disease<br>☐ Southern Tick Associated Rash Illness<br>☐ Alpha-Gal Allergy (e.g., Red Meat Allergy)<br>☐ Anaplasmosis<br>☐ Other<br>(Check all that apply)
If you were diagnosed with a different tick-borne disease, please list here:	__________________________________
When were you diagnosed?	__________________________<br>(Approximate Date)
Did you donate blood in the 30 days prior to your symptom onset?	◯ Yes<br>◯ No<br>◯ Unknown
Date of blood donation:	__________________________
Were you ever contacted by health authorities about transfusion-associated infections related to your blood donation?	◯ Yes<br>◯ No<br>◯ Unknown

Household Information

How many people are in your household/live with you (including yourself)?	________________________
How many children aged 5-18 years old live with you?	________________________
How many children younger than 5 years old live with you?	________________________
Which of the following applies to you?	◯ I live in a single-family home that I own.<br>◯ I live in a multi-family property (like a townhome or duplex) and I own my unit.<br>◯ I live in a single-family home that I rent.<br>◯ I live in a multi-family property (like a townhome or duplex) that I rent.<br>◯ I live in an apartment that I rent.<br>◯ I have another living situation.
What is your current living situation?	______________________________
Approximately how many square feet is your living space?	◯ < 500 sq feet (< 46.5 sq m)<br>◯ 500-1000 sq feet (46.5-93 sq m)<br>◯ 1000-2000 sq feet (93-186 sq m)<br>◯ >2000 sq feet (>186 sq m)<br>◯ Not sure<br>◯ N/A

Tick Exposure History

Before your recent medical visit, did you find a tick on you?	◯ Yes<br>◯ No

Below is an image of different stages of the tick life cycle.

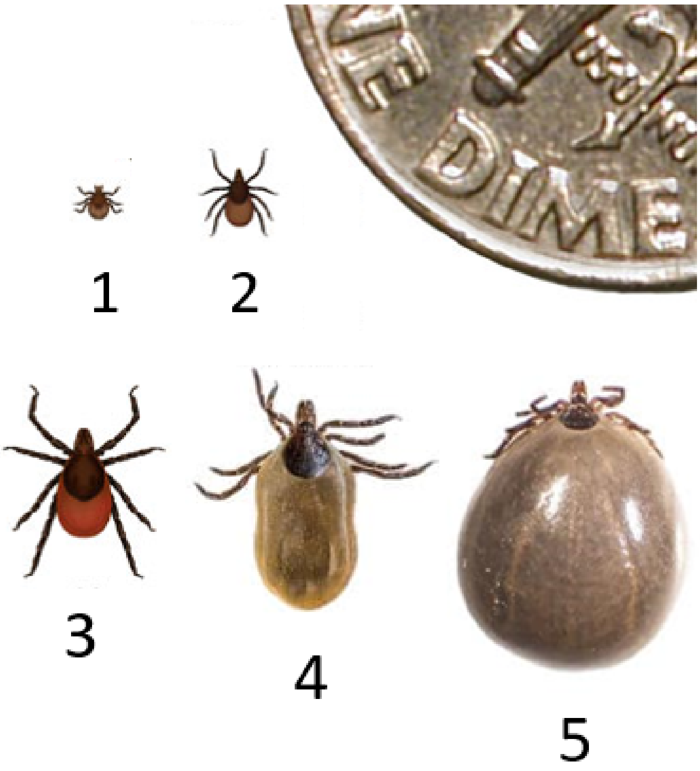

In the image above, which number best matches the type of tick you found on you?	◯ 1<br>◯ 2<br>◯ 3<br>◯ 4<br>◯ 5<br>◯ Not sure

Below is an image of different species of ticks.

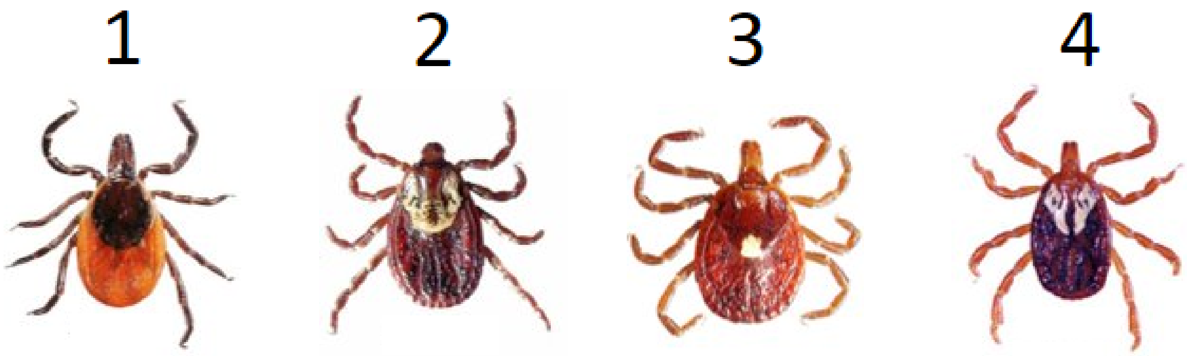

Which number above best matches the tick you found on you?	◯ 1<br>◯ 2<br>◯ 3<br>◯ 4<br>◯ Not sure
Please describe in words the tick you found on yourself:	__________________________________
Please upload a picture of the tick here if you took one.	
Where do you think you would have been exposed to a tick?	__________________________________
Have you found a tick on you in the past 3 years?	◯ Yes ◯ No
Did you ever receive preventative antibiotics from your doctor after any of these tick bites?	◯ Yes<br>◯ No<br>◯ Don't know/Not sure
What antibiotic did you take after being bitten by a tick during this time period? Specify name, dose, and duration.	__________________________________
How frequently you find ticks on you?	◯ Rarely<br>◯ Once a week<br>◯ Once a month<br>◯ Once a year
Do you have any house pets that spend at least some time outside? For the purposes of this study, a house pet is an animal such as a cat or dog that is allowed to spend some time inside your home between May and October.	◯ Yes<br>◯ No<br>◯ Don't know/Not sure
What kinds of pets do you have that spend time outside?	☐ Dog<br>☐ Cat<br>☐ Rabbit<br>☐ Other
If other, please include species name and number	____________________________
How many dogs do you have?	____________________________
In the past 6 months, have you used any product to control ticks on your dog(s)?	◯ Yes<br>◯ No

What type of tick control product do you use on your dog(s)?

☐ Powder
☐ Collar
☐ Spray
☐ Topical spot
☐ Bath
☐ Other
☐ Don't know/Not sure

For other types of products, please specify:

____

Approximately when did you last treat your dog(s)?

____
(Approximate date)

How many cats do you have?

____

In 2021, have you used any product to control ticks on your cat(s)?

◯ Yes
◯ No

What type of tick control product do you use on your cat(s)?

☐ Powder
☐ Collar
☐ Spray
☐ Topical spot
☐ Bath
☐ Other
☐ Don't know/Not sure

For other types of products, please specify:

____

Approximately when did you last treat your cat(s)?

____
(Approximate date)

How many rabbits do you have?

____

In 2021, have you used any product to control ticks on your rabbit(s)?

◯ Yes
◯ No

What type of tick control product have you used on your rabbit(s) this year?

☐ Powder
☐ Collar
☐ Spray
☐ Topical spot
☐ Bath
☐ Other
☐ Don't know/Not sure

Please specify:

____

Approximately when did you last treat your rabbit(s)?

____
(Approximate date)

What kind(s) of other pets do you have and how many? __________________________________

During current year have you used any product to control ticks on your other pet(s)?
- ◯ Yes
- ◯ No

In 2021, have you used any product to control ticks on your other pet(s)?
- ☐ Powder
- ☐ Collar
- ☐ Spray
- ☐ Topical spot
- ☐ Bath
- ☐ Other
- ☐ Don't know/Not sure

For other products, please specify type: ____________________________

Approximately when did you last treat your other pet(s)? ____________________________
(Approximate date)

Do you check your pets for ticks?
- ◯ No
- ◯ Yes, all of them
- ◯ Yes, some of them

Which of your pets do you check for ticks? __________________________________

OUTDOOR ANIMALS

Do you keep any outdoor pets or livestock in your yard? Here we are referring to livestock or animals that are purely outdoor and do not come inside the house.
- ◯ Yes
- ◯ No
- ◯ Don't know/Not sure

What kind of outdoor pets and livestock do you have in your yard?
- ☐ Cats
- ☐ Dogs
- ☐ Rabbits
- ☐ Horses
- ☐ Goats
- ☐ Guinea Fowl
- ☐ Other

How many dogs? ____________________________

How many cats? ____________________________

How many rabbits? ____________________________

How many horses? ____________________________

How many goats?

____

How many guinea fowl?

____

Please specify the other type(s) of outdoor animal(s):

____

How many of these animals do you have?

____

YARD/LAWN

Do you live on a property that has a yard, lawn, or woods?

◯ Yes
◯ No

What is the approximate size of the property you live on?

◯ Less than 0.5 acre
◯ 0.5 - 1 acre
◯ 1 - 5 acres
◯ 5 - 10 acres
◯ More than 10 acres
◯ Don't know / Not sure

How much of the property is lawn/grass?

◯ All of it
◯ Greater than half of it
◯ About half of it
◯ Less than half of it
◯ No lawn on property
◯ Don't Know/Not Sure

How much of your property is wooded?

◯ All of it
◯ Greater than half of it
◯ About half of it
◯ Less than half of it
◯ No lawn on property
◯ Don't Know/Not Sure

In the one month prior to your illness, how frequently was your lawn cut?

◯ Not cut during this time
◯ Once
◯ Twice
◯ Three times
◯ Four times
◯ More than four times
◯ Don't know/Not sure

Does your yard border on woods or brushy areas including property not owned by you or your landlord?

◯ Yes
◯ No
◯ Don't know/Not sure

How much of the property borders woods or forest?

◯ All of it
◯ Greater than half of it
◯ About half of it
◯ Less than half of it
◯ No woods on property
◯ Don't Know/Not Sure

Do tree branches or brush from the woods overhang the lawn?	◯ Yes<br>◯ No<br>◯ Don't know/Not sure
Do you have a vegetable garden in your yard?	◯ Yes<br>◯ No<br>◯ Don't know/Not sure
Does your yard have fencing around it or parts of it?	◯ Yes<br>◯ No<br>◯ Don't know/Not sure
Do you have a fence that completely surrounds a portion of your yard on all sides? (Does not apply to fenced gardens.)	◯ Yes<br>◯ No<br>◯ Don't know/Not sure
What is the approximate height of the fencing?	◯ < 5 ft.<br>◯ Between 5 and 6 ft.<br>◯ Between 6 and 7 ft.<br>◯ >8 ft.<br>◯ Don't know/Not sure
How would you describe your fence construction?	◯ Solid surface<br>◯ Chain-link or wire<br>◯ Picket style<br>◯ Split-rail<br>◯ Other<br>◯ Don't know/Not sure
For other construction types, please specify:	__________________________
Approximately how much land is enclosed by your fence?	__________________________
Have pesticides ever been applied on your yard specifically to control ticks?	◯ Yes<br>◯ No<br>◯ Don't know/Not sure
Prior to your illness, when was the last time that pesticide was applied?	◯ In the month before my illness<br>◯ 1 month ago<br>◯ 2 months ago<br>◯ 3+ months ago<br>◯ Don't know/Not sure
Who last applied the pesticide on your yard?	◯ Self<br>◯ Other person in home<br>◯ Landlord/other property owner<br>◯ Professional company<br>◯ Other<br>◯ Don't know/Not sure
For others, please specify who:	__________________________

What was the chemical used in the pesticide?	◯ Bifenthrin<br>◯ Cyfluthrin<br>◯ Deltamethrin<br>◯ Permethrin<br>◯ lambda-Cyhalothrin<br>◯ Carbaryl<br>◯ Pyrethrin<br>◯ Natural product (e.g., cedar oil)<br>◯ Other<br>◯ Don't know/ Not sure
Please specify the chemical used:	__________________________________
When pesticides were last applied to your yard to control ticks, where was the pesticide applied?	◯ Entire yard including lawn, brush, woods, ornamental plants, etc.<br>◯ Entire lawn<br>◯ Perimeter of lawn<br>◯ Ornamentals<br>◯ Along dry barrier<br>◯ Other<br>◯ Don't know/Not sure
Have any pesticides other than tick-control pesticides been applied to your yard during this calendar year?	◯ Yes<br>◯ No<br>◯ Don't know/Not sure
Who last applied this other pesticide on your yard?	◯ Self<br>◯ Other person in home<br>◯ Homeowner if property not owned by subject<br>◯ Professional company<br>◯ Other<br>◯ Don't know/Not sure
For others, please specify who:	__________________________________
Please specify the following details about this other pesticide(s):<br><br>Brand names<br>Formulation spray, granules, etc.<br>For control of what kind of pests?	____
Do you use any rodent-targeted tick-control devices in your yard such as bait boxes or Damminix?	◯ Yes<br>◯ No<br>◯ Don't know/Not sure
What kind of rodent-targeted tick-control devices have been used in your yard? [Select all that apply]	☐ Damminix Tubes<br>☐ Bait Box MaxForceTM, e.g.<br>☐ Other<br>☐ Don't know/Not sure
Please specify the other type of rodent-targeted tick-control device:	__________________________________

Prior to your illness, what was the last time that rodent-targeted tick-control devices were set on your yard?	◯ In the month before my illness<br>◯ 1 month ago<br>◯ 2 months ago<br>◯ 3+ months ago<br>◯ Don't know/Not sure
How long before onset of your recent illness had you been using rodent-targeted tick-control devices on your yard?	◯ < 1 year<br>◯ 1-2 years<br>◯ >2 years<br>◯ Don't know/Not sure
Who last set the rodent-control devices on your yard?	◯ Self<br>◯ Other person in home<br>◯ Homeowner if property not owned by subject<br>◯ Professional company<br>◯ Other<br>◯ Don't know/Not sure
Please specify who placed the device(s):	__________________________________

POTENTIAL SMALL ANIMAL HABITATS

During the last two years, have you had birdfeeders in your yard?	◯ Yes<br>◯ No<br>◯ Don't know/Not sure
Have birdfeeders been used since April 1st of 2021?	◯ Yes<br>◯ No<br>◯ Don't know/Not sure
How many feet from your house is your nearest birdfeeder located?	◯ < 10 ft.<br>◯ 10-25 ft.<br>◯ 26-50 ft.<br>◯ >50 ft.<br>◯ Don't know/Not sure
Over what type of surface is your nearest birdfeeder located?	◯ Lawn<br>◯ Garden or flower bed<br>◯ Leaf litter<br>◯ Gravel<br>◯ Wood chips or mulch<br>◯ Other<br>◯ Don't know/Not sure
Other (Please specify):	__________________________________
Do you have a log pile on your yard?	◯ Yes<br>◯ No<br>◯ Don't know/Not sure
How many feet from your house is your nearest log pile?	◯ < 10 ft.<br>◯ 10-25 ft.<br>◯ 26-50 ft.<br>◯ >50 ft.<br>◯ Don't know/Not sure

Personal Protection

TIME OUTSIDE

In the one month prior to your illness, did you spend any time outside in your yard? (Not counting time spent only on the porch, patio, or deck.)
- ◯ Yes
- ◯ No
- ◯ Don't know/Not sure

The next two questions pertain to spending time in your yard during the week, which only refers to days in the normal work week-Monday through Friday.

On average, in the one month prior to your illness, how many times did you go into your yard during the week?
____
(Times per week)

On average, in the one month prior to your illness approximately how many hours per week did you spend in your yard? (Not including spending time on the porch/patio or deck.)
____
(Hours per week)

The next two questions pertain to spending time in your yard during the weekend, which only refers to Saturdays and Sundays.

On average, in the one month prior to your illness, how many times you go into your yard on the weekend?
____
(Times per weekend)

On average, in the one month prior to your illness, approximately how many hours on the weekend did you spend in your yard?
____
(Hours per weekend)

REPELLENT USE

In the one month prior to your illness, did you ever use insect repellent when spending time outside in your yard?
- ◯ Yes
- ◯ No
- ◯ Don't know/Not sure

When spending time outside in your yard between in the one month prior to your illness, how often would you say that you used insect repellent?
- ◯ All of the time
- ◯ More than half the time
- ◯ About half the time
- ◯ Less than half the time
- ◯ Don't know/Not sure

Do you know the brand name of the insect repellent that you used while spending time in your yard?
- ◯ Yes
- ◯ No

What is the brand name of the insect repellent that you/your child most frequently used during this time period?
____

Does the repellent used contain DEET?
- ◯ Yes
- ◯ No
- ◯ Don't know/Not sure

In the month prior to your illness, did you wear clothing that is treated with permethrin insecticide when spending time in your yard?	◯ Yes<br>◯ No<br>◯ Don't know/Not sure
In the one month prior to your illness, how frequently did you wear long pants when spending time outside in your yard?	◯ All of the time<br>◯ More than half the time<br>◯ About half the time<br>◯ Less than half the time<br>◯ Never<br>◯ Don't know/Not sure
When you did wear long pants outside in your yard, how often were pant legs tucked into socks?	◯ All of the time<br>◯ More than half the time<br>◯ About half the time<br>◯ Less than half the time<br>◯ Never<br>◯ Don't know/Not sure
In the one month prior to your illness, how frequently did you wear light-colored clothing when spending time outside in your yard?	◯ All of the time<br>◯ More than half the time<br>◯ About half the time<br>◯ Less than half the time<br>◯ Never<br>◯ Don't know/Not sure
In the month prior to your illness how often did you shower/bathe within 2 hours after returning indoors?	◯ All of the time<br>◯ More than half the time<br>◯ About half the time<br>◯ Less than half the time<br>◯ Never<br>◯ Don't know/Not sure

CHECKING FOR TICKS

In the month prior to your illness, how frequently did you check yourself for ticks within 36 hours after spending time outside in your yard?	◯ All of the time<br>◯ More than half the time<br>◯ About half the time<br>◯ Less than half the time<br>◯ Never<br>◯ Don't know/Not sure
When did you perform tick checks after you spent time outdoors in your yard?	◯ Within 12 hours after spending time outdoors<br>◯ Within 24 hours after spending time outdoors<br>◯ Within 36 hours after spending time outdoors<br>◯ Don't know/Not sure

OTHER:

In the one month prior to your illness, did you participate in recreational activities that may have exposed you to ticks? This includes only activities performed outside of the yard, such as trail running, camping, Garden Club, hiking, etc.	◯ Yes<br>◯ No<br>◯ Don't know/Not sure
Please specify the activities:	__________________________________

In the one month prior to your illness, did you travel to any locations that may have exposed you to ticks?	◯ Yes<br>◯ No<br>◯ Don't know/Not sure
Please specify the location(s) of travel:	__________________________________
