# Supplementary material for: Enhanced surveillance for tick-borne rickettsiosis and ehrlichiosis in North Carolina: Protocol and preliminary results
Source: PLoS One. 2025 May 12;20(5):e0320361. doi: 10.1371/journal.pone.0320361 (PMC12068726; doi:10.1371/journal.pone.0320361)
Supplement: S9 File — (PDF) [file pone.0320361.s009.pdf]

60 Day Follow-Up Survey

Please complete the survey below.Thank you!

SYMPTOMS (SÍNTOMAS)

Please indicate your experience OVER THE PAST 2 WEEKS with these symptoms:
(Indique su experiencia con los siguientes síntomas EN LAS ÚLTIMAS 2 SEMANAS:)

	None	Mild	Moderate	Severe
Joint swelling	◯	◯	◯	◯
Double vision	◯	◯	◯	◯
Drooping facial muscle	◯	◯	◯	◯
Drooping eyelid (s)	◯	◯	◯	◯
Tinnitus (ringing in the ear)	◯	◯	◯	◯
Heart palpitations (fast-beating, fluttering, pounding heart)	◯	◯	◯	◯
Swollen lymph nodes	◯	◯	◯	◯
Vomiting	◯	◯	◯	◯
Diarrhea	◯	◯	◯	◯
Sore throat	◯	◯	◯	◯
Anxiety	◯	◯	◯	◯
Fatigue	◯	◯	◯	◯
Joint pain	◯	◯	◯	◯
Difficulty focusing/concentrating	◯	◯	◯	◯
Muscle pain	◯	◯	◯	◯
Memory problems	◯	◯	◯	◯
Difficulty finding words	◯	◯	◯	◯
Sleep problems	◯	◯	◯	◯
Neck pain	◯	◯	◯	◯
Abnormal sensation of the skin (tingling, pricking, chilling, burning, numbness) (Hands/Feet)	◯	◯	◯	◯
Abnormal sensation of the skin (tingling, pricking, chilling, burning, numbness) (Face/Scalp)	◯	◯	◯	◯
Irritability	◯	◯	◯	◯
Low back pain	◯	◯	◯	◯
Headache	◯	◯	◯	◯
Photophobia (light sensitivity or intolerance)	◯	◯	◯	◯
Dizziness	◯	◯	◯	◯

Visual clarity problems	○	○	○	○
Chills	○	○	○	○
Coordination problems	○	○	○	○
Sweats	○	○	○	○
Fasciculation (muscle twitch)	○	○	○	○
Depression	○	○	○	○
Breathing difficulty	○	○	○	○
Urination changes	○	○	○	○
Nausea	○	○	○	○
Fever	○	○	○	○

FATIGUE SEVERITY SCALE

The next section evaluates fatigue. Please select the number between 1 and 7 which you feel best fits the following statements. This refers to your usual way of life WITHIN THE LAST WEEK. 1 indicates "strongly agree" and 7 indicates "strongly disagree."

	1 - Strongly Agree	2	3	4	5	6	7 - Strongly Disagree
1. My motivation is lower when I am fatigued.	○	○	○	○	○	○	○
2. Exercise brings on my fatigue.	○	○	○	○	○	○	○
3. I am easily fatigued.	○	○	○	○	○	○	○
4. Fatigue interferes with my physical functioning.	○	○	○	○	○	○	○
5. Fatigue causes frequent problems for me.	○	○	○	○	○	○	○
6. My fatigue prevents sustained physical functioning.	○	○	○	○	○	○	○
7. Fatigue interferes with carrying out certain duties and responsibilities.	○	○	○	○	○	○	○
8. Fatigue is among my most disabling symptoms.	○	○	○	○	○	○	○
9. Fatigue interferes with my work, family, or social life.	○	○	○	○	○	○	○

Please select the number which describes your overall fatigue with 0 being normal and 10 being the worst.

○ 0 - Normal
○ 1
○ 2
○ 3
○ 4
○ 5
○ 6
○ 7
○ 8
○ 9
○ 10 - Worst

MCGILL PAIN QUESTIONAIRRE

Please rate the severity of the below types of pain in the last 4 weeks.

	None	Mild	Moderate	Severe
Throbbing	○	○	○	○
Shooting	○	○	○	○
Stabbing	○	○	○	○
Sharp	○	○	○	○
Cramping	○	○	○	○
Gnawing	○	○	○	○
Hot-Burning	○	○	○	○
Aching	○	○	○	○
Heavy	○	○	○	○
Tender	○	○	○	○
Splitting	○	○	○	○
Tiring-Exhausting	○	○	○	○
Sickening	○	○	○	○
Fearful	○	○	○	○
Punishing-Cruel	○	○	○	○

SLEEP QUALITY

The following questions relate to your usual sleep habits during the past 4 weeks only. Your answers should indicate the most accurate reply for the majority of days and nights in the past 4 weeks.

1. During the past 4 weeks, what time have you usually gone to bed at night?

____
(BED TIME)

2. During the past 4 weeks, how long (in minutes) has it usually taken you to fall asleep each night?

____
(NUMBER OF MINUTES)

3. During the past 4 weeks, what time have you usually gotten up in the morning?

____
(GETTING UP TIME)

4. During the past 4 weeks, how many hours of actual sleep did you get at night? (This may be different than the number of hours you spent in bed.)

____
(hours of sleep per night)

For each of the remaining questions, check the one best response. Please answer all questions.

5. During the past 4 weeks, how often have you had trouble sleeping because you . . .

	Not during the past month	Less than once a week	Once or twice a week	Three or more times a week
a) Cannot get to sleep within 30 minutes	◯	◯	◯	◯
b) Wake up in the middle of the night or early morning	◯	◯	◯	◯
c) Have to get up to use the bathroom	◯	◯	◯	◯
d) Cannot breathe comfortably	◯	◯	◯	◯
e) Cough or snore loudly	◯	◯	◯	◯
f) Feel too cold	◯	◯	◯	◯
g) Feel too hot	◯	◯	◯	◯
h) Had bad dreams	◯	◯	◯	◯
i) Have pain	◯	◯	◯	◯

j) Any other reason(s)?
◯ Yes
◯ No

Please describe ______________________

How often during the past 4 weeks have you had trouble sleeping because of this?
◯ Not during the past month
◯ Less than once a week
◯ Once or twice a week
◯ Three or more times a week

6. During the past 4 weeks, how would you rate your sleep quality overall?
◯ Very good
◯ Fairly good
◯ Fairly bad
◯ Very bad

7. During the past month, how often have you taken medicine to help you sleep (prescribed or "over the counter")?
◯ Not during the past month
◯ Less than once a week
◯ Once or twice a week
◯ Three or more times a week

8. During the past month, how often have you had trouble staying awake while driving, eating meals, or engaging in social activity?
◯ Not during the past month
◯ Less than once a week
◯ Once or twice a week
◯ Three or more times a week

9. During the past 4 weeks, how much of a problem has it been for you to keep up enough enthusiasm to get things done?
◯ No problem at all
◯ Only a very slight problem
◯ Somewhat of a problem
◯ A very big problem

10. Do you have a bed partner or room mate?

◯ No bed partner or room mate
◯ Partner/room mate in other room
◯ Partner in same room, but not same bed
◯ Partner in same bed

If you have a roommate or bed partner, ask them how often in the past 4 weeks you have had:

	Not during the past month	Less than once a week	Once or twice a week	Three or more times a week
a) Loud snoring	◯	◯	◯	◯
b) Long pauses between breaths while asleep	◯	◯	◯	◯
c) Legs twitching or jerking while you sleep	◯	◯	◯	◯
d) Episodes of disorientation or confusion during sleep	◯	◯	◯	◯

e) Other restlessness while you sleep?

◯ Yes
◯ No

Please describe

____

How often?

◯ Not during the past month
◯ Less than once a week
◯ Once or twice a week
◯ Three or more times a week

BECK'S DEPRESSION INVENTORY

For each question in the next section, please choose the statement that most applies to you today.

1

◯ I do not feel sad.
◯ I feel sad
◯ I am sad all the time and I can't snap out of it.
◯ I am so sad and unhappy that I can't stand it.

2

◯ I am not particularly discouraged about the future.
◯ I feel discouraged about the future.
◯ I feel I have nothing to look forward to.
◯ I feel the future is hopeless and that things cannot improve.

3

◯ I do not feel like a failure.
◯ I feel I have failed more than the average person.
◯ As I look back on my life, all I can see is a lot of failures.
◯ I feel I am a complete failure as a person.

4	◯ I get as much satisfaction out of things as I used to.<br>◯ I don't enjoy things the way I used to.<br>◯ I don't get real satisfaction out of anything anymore.<br>◯ I am dissatisfied or bored with everything.
5	◯ I don't feel particularly guilty<br>◯ I feel guilty a good part of the time.<br>◯ I feel quite guilty most of the time.<br>◯ I feel guilty all of the time.
6	◯ I don't feel I am being punished.<br>◯ I feel I may be punished.<br>◯ I expect to be punished.<br>◯ I feel I am being punished.
7	◯ I don't feel disappointed in myself.<br>◯ I am disappointed in myself.<br>◯ I am disgusted with myself.<br>◯ I hate myself.
8	◯ I don't feel I am any worse than anybody else.<br>◯ I am critical of myself for my weaknesses or mistakes.<br>◯ I blame myself all the time for my faults.<br>◯ I blame myself for everything bad that happens.
9	◯ I don't have any thoughts of killing myself.<br>◯ I have thoughts of killing myself, but I would not carry them out.<br>◯ I would like to kill myself.<br>◯ I would kill myself if I had the chance.
10	◯ I don't cry any more than usual.<br>◯ I cry more now than I used to.<br>◯ I cry all the time now.<br>◯ I used to be able to cry, but now I can't cry even though I want to.
11	◯ I am no more irritated by things than I ever was.<br>◯ I am slightly more irritated now than usual.<br>◯ I am quite annoyed or irritated a good deal of the time.<br>◯ I feel irritated all the time.
12	◯ I have not lost interest in other people.<br>◯ I am less interested in other people than I used to be.<br>◯ I have lost most of my interest in other people.<br>◯ I have lost all of my interest in other people.
13	◯ I make decisions about as well as I ever could.<br>◯ I put off making decisions more than I used to.<br>◯ I have greater difficulty in making decisions more than I used to.<br>◯ I can't make decisions at all anymore.

14	◯ I don't feel that I look any worse than I used to.<br>◯ I am worried that I am looking old or unattractive.<br>◯ I feel there are permanent changes in my appearance that make me look unattractive<br>◯ I believe that I look ugly.
15	◯ I can work about as well as before.<br>◯ It takes an extra effort to get started at doing something.<br>◯ I have to push myself very hard to do anything.<br>◯ I can't do any work at all.
16	◯ I can sleep as well as usual.<br>◯ I don't sleep as well as I used to.<br>◯ I wake up 1-2 hours earlier than usual and find it hard to get back to sleep.<br>◯ I wake up several hours earlier than I used to and cannot get back to sleep.
17	◯ I don't get more tired than usual.<br>◯ I get tired more easily than I used to.<br>◯ I get tired from doing almost anything.<br>◯ I am too tired to do anything.
18	◯ My appetite is no worse than usual.<br>◯ My appetite is not as good as it used to be.<br>◯ My appetite is much worse now.<br>◯ I have no appetite at all anymore.
19	◯ I haven't lost much weight, if any, lately.<br>◯ I have lost more than five pounds.<br>◯ I have lost more than ten pounds.<br>◯ I have lost more than fifteen pounds.
20	◯ I am no more worried about my health than usual.<br>◯ I am worried about physical problems like aches, pains, upset stomach, or constipation.<br>◯ I am very worried about physical problems and it's hard to think of much else.<br>◯ I am so worried about my physical problems that I cannot think of anything else.
21	◯ I have not noticed any recent change in my interest in sex.<br>◯ I am less interested in sex than I used to be.<br>◯ I have almost no interest in sex.<br>◯ I have lost interest in sex completely.
Total Score	______________________<br>(Levels of Depression)

SF-36 QUESTIONNAIRE ON GENERAL HEALTH

In general, would you say your health is:

- ◯ Excellent
- ◯ Very Good
- ◯ Good
- ◯ Fair
- ◯ Poor

Compared to one year ago, how would you rate your health in general now?

- ◯ Much better now than one year ago
- ◯ Somewhat better now than one year ago
- ◯ About the same
- ◯ Somewhat worse now than one year ago
- ◯ Much worse than one year ago

LIMITATIONS OF ACTIVITIES:

The following items are about activities you might do during a typical day. Does your health now limit you in these activities? If so, how much?

	Yes, Limited a Lot	Yes, Limited a Little	No, Not Limited at all
Vigorous activities, such as running, lifting heavy objects, participating in strenuous sports.	◯	◯	◯
Moderate activities, such as moving a table, pushing a vacuum cleaner, bowling, or playing golf	◯	◯	◯
Lifting or carrying groceries	◯	◯	◯
Climbing several flights of stairs	◯	◯	◯
Climbing one flight of stairs	◯	◯	◯
Bending, kneeling, or stooping	◯	◯	◯
Walking more than a mile	◯	◯	◯
Walking several blocks	◯	◯	◯
Walking one block	◯	◯	◯
Bathing or dressing yourself	◯	◯	◯

PHYSICAL HEALTH PROBLEMS:

During the past 4 weeks, have you had any of the following problems with your work or other regular daily activities as a result of your physical health?

	Yes	No
Cut down the amount of time you spent on work or other activities	◯	◯
Accomplished less than you would like	◯	◯
Were limited in the kind of work or other activities	◯	◯

Had difficulty performing the work or other activities (for example, it took extra effort)	◯	◯

During the past 4 weeks, how much of the time has your physical health interfered with your social activities (like visiting with friends, relatives, etc.)?

- ◯ All of the time
- ◯ Most of the time
- ◯ Some of the time
- ◯ A little bit of the time
- ◯ None of the time

During the past 4 weeks, how much did pain interfere with your normal work (including both work outside the home and housework)?

- ◯ Not at all
- ◯ A little bit
- ◯ Moderately
- ◯ Quite a bit
- ◯ Extremely

How much bodily pain have you had during the past 4 weeks?

- ◯ None
- ◯ Very Mild
- ◯ Mild
- ◯ Moderate
- ◯ Severe
- ◯ Very Severe

EMOTIONAL HEALTH PROBLEMS:

During the past 4 weeks, have you had any of the following problems with your work or other regular daily activities as a result of any emotional problems (such as feeling depressed or anxious)?

	Yes	No
Cut down the amount of time you spent on work or other activities	◯	◯
Accomplished less than you would like	◯	◯
Didn't do work or other activities as carefully as usual	◯	◯

How much have emotional problems interfered with your social activities (like visiting with friends, relatives, etc.)?

- ◯ Not at all
- ◯ Slightly
- ◯ Moderately
- ◯ Severe
- ◯ Very Severe

ENERGY AND EMOTIONS:

These questions are about how you feel and how things have been with you during the last 4 weeks. For each question, please give the answer that comes closest to the way you have been feeling.

	None of the time	A little bit of the time	Some of the time	A good bit of the time	Most of the time	All of the time

Did you feel full of pep?	○	○	○	○	○	○
Have you been a very nervous person?	○	○	○	○	○	○
Have you felt so down in the dumps that nothing could cheer you up?	○	○	○	○	○	○
Have you felt calm and peaceful?	○	○	○	○	○	○
Did you have a lot of energy?	○	○	○	○	○	○
Have you felt downhearted and blue?	○	○	○	○	○	○
Did you feel worn out?	○	○	○	○	○	○
Have you been a happy person?	○	○	○	○	○	○
Did you feel tired?	○	○	○	○	○	○

GENERAL HEALTH:

How true or false is each of the following statements for you?

	Definitely true	Mostly true	Don't know	Mostly false	Definitely false
I seem to get sick a little easier than other people	○	○	○	○	○
I am as healthy as anybody I know	○	○	○	○	○
I expect my health to get worse	○	○	○	○	○
My health is excellent	○	○	○	○	○
